# Correction: A FRET-based biosensor for measuring Gα13 activation in single cells

**DOI:** 10.1371/journal.pone.0195649

**Published:** 2018-04-04

**Authors:** Marieke Mastop, Nathalie R. Reinhard, Cristiane R. Zuconelli, Fenna Terwey, Theodorus W. J. Gadella, Jakobus van Unen, Merel J. W. Adjobo-Hermans, Joachim Goedhart

In the Funding section, the grant from the funder CAPES (Coordenação de Aperfeiçoamento Pessoal de Nível Superior) is attributed incorrectly. The correct recipient for this grant is CRZ.

The correct funding information is as follows: JG was supported by a NWO Chemical Sciences ECHO grant (711.013.009). CRZ was supported financially by the Brazilian research funding agency CAPES (Coordenação de Aperfeiçoamento Pessoal de Nível Superior) (grant: BEX 13247/13-1). The funders had no role in study design, data collection and analysis, decision to publish, or preparation of the manuscript.
