# Supplementary figures and images for: A FRET-based biosensor for measuring Gα13 activation in single cells
Source: PLoS One. 2018 Mar 5;13(3):e0193705. doi: 10.1371/journal.pone.0193705 (PMC5837189; doi:10.1371/journal.pone.0193705)

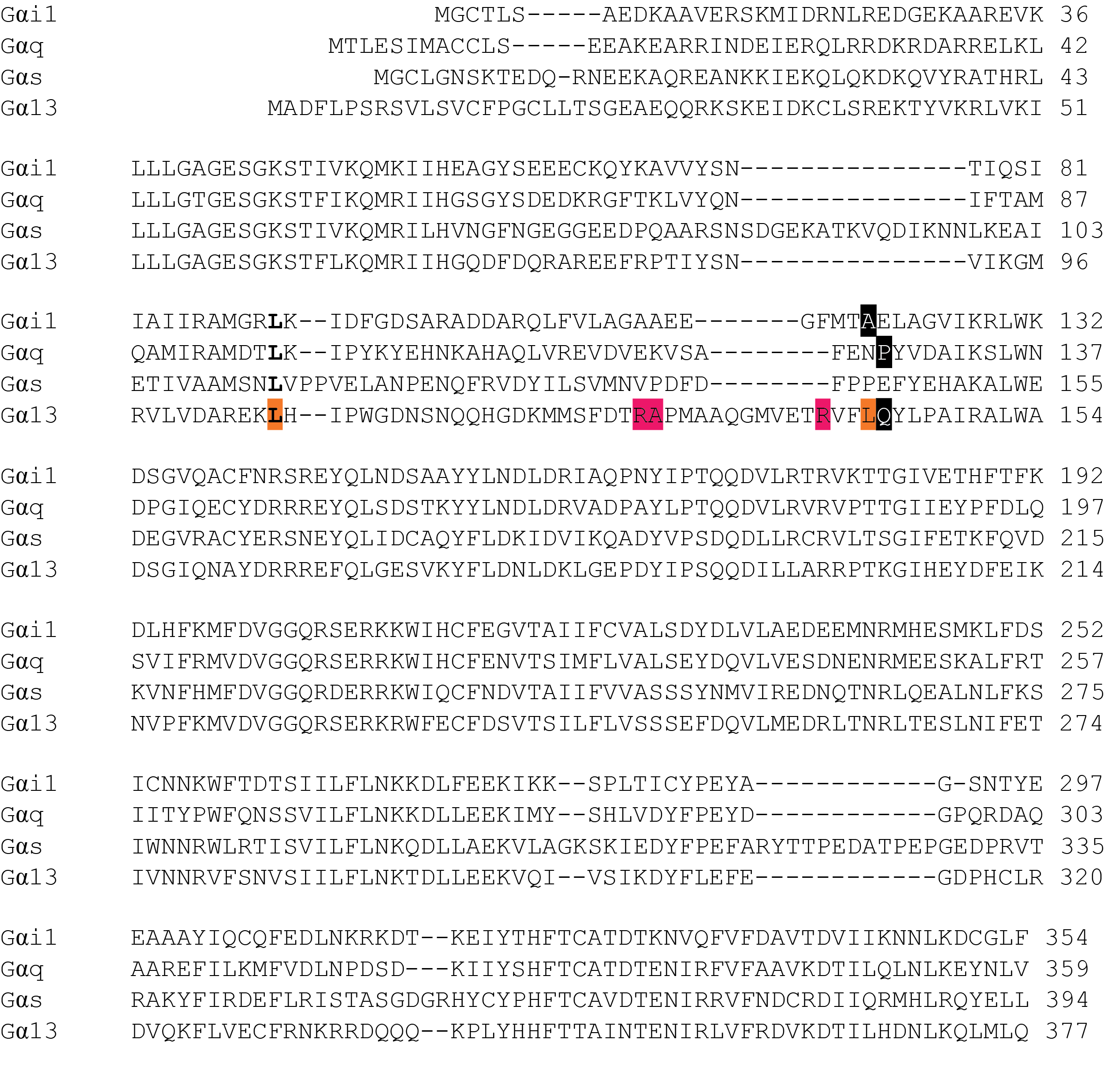

Supplement: S1 Fig — Note that the amino acid sequence of Gαs is that of the short isoform. The highlighted residues indicate the amino acid preceding the inserted fluorescent protein (or luciferase). In bold, the sites that were previously used to insert Rluc (Saulière et al., 2012). Insertion of mTurquoise2-Δ9 in Gα13 after residue Q144 (black) was based on homology with previous insertions in Gαq and Gαi (black). Successful sites for inserting mTurquoise2-Δ9 (R128, A129 and R140) in pink and unsuccessful sites (L106 and L143) in orange. (PNG) [file pone.0193705.s001.png]

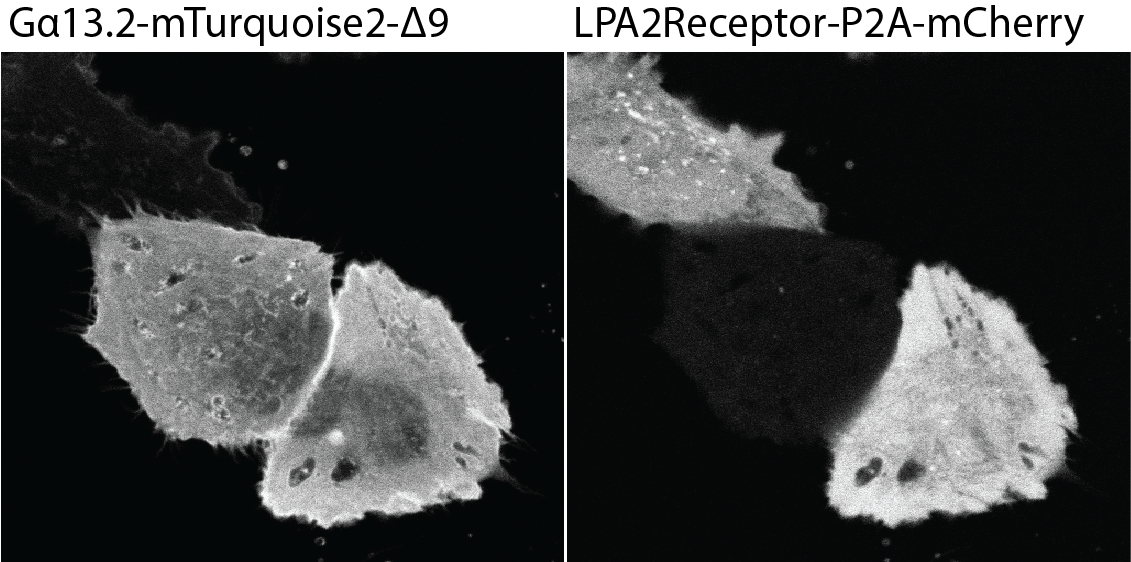

Supplement: S2 Fig — The width of the images is 67μm. (PNG) [file pone.0193705.s002.png]

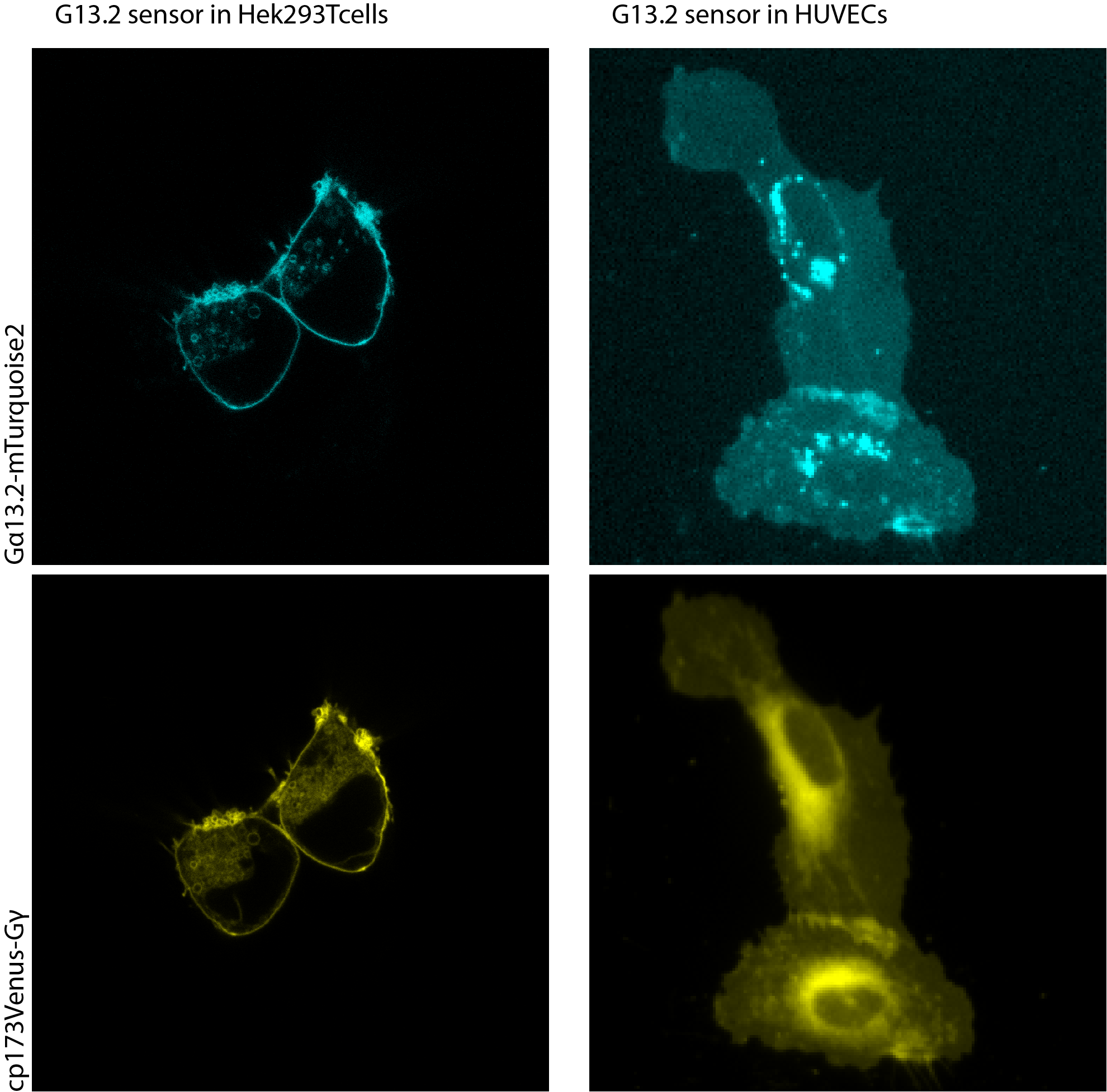

Supplement: S3 Fig — The upper images show Gα13.2 localization and the lower images show Gγ localization. Hek293T cells were excited with 457nm (CFP) and 514nm (YFP) light and respectively, a 482/35BP and 540/30BP were used for detection of emission light. The width of the images is 70μm. HUVEC cells were excited with 420nm (CFP) and 490nm (YFP) light and respectively, a 470/30BP and 535/30BP were used for detection of emission light. (PNG) [file pone.0193705.s003.png]

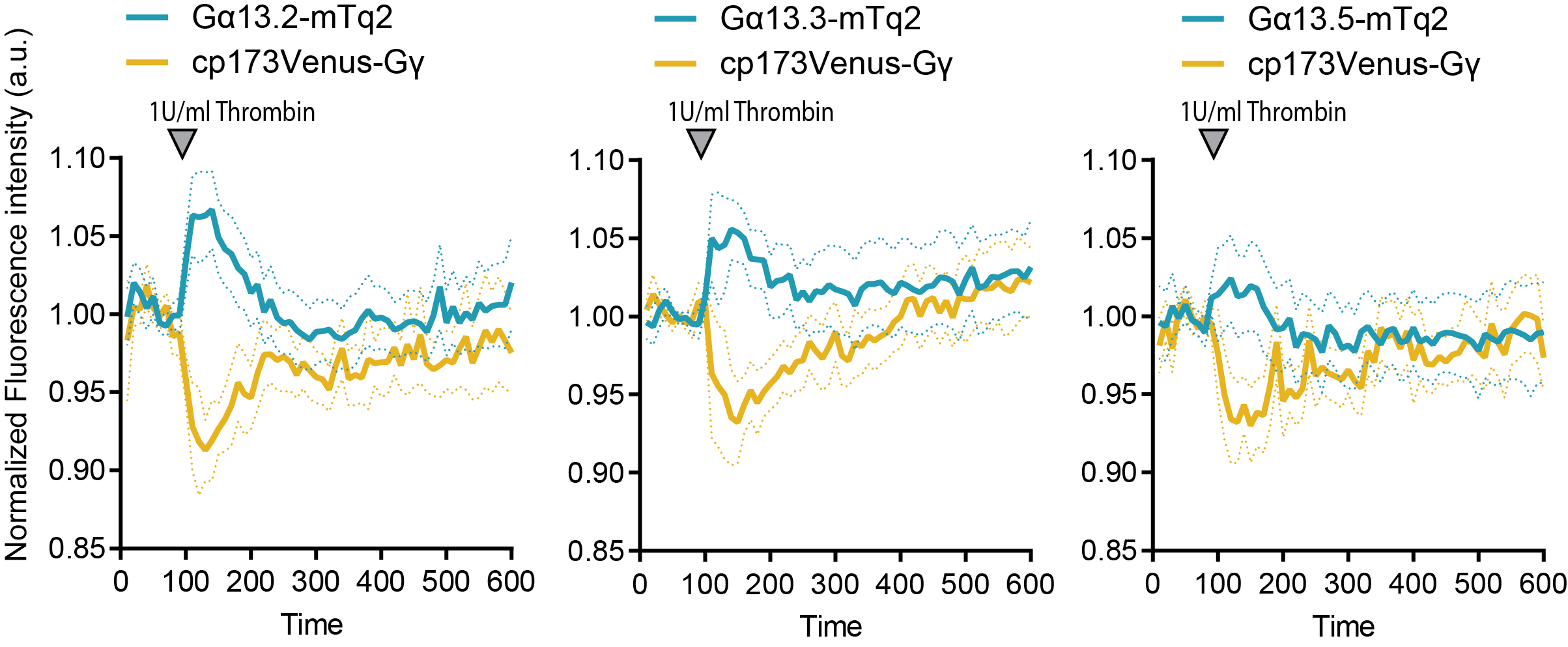

Supplement: S4 Fig — The number of cells analyzed is: G13.2 sensor n = 16, G13.3 sensor n = 11, G13.5 sensor n = 16. (PNG) [file pone.0193705.s004.png]
